# The link between smoking, emphysema, and fibrosis: A retrospective cohort study

**DOI:** 10.18332/tid/190689

**Published:** 2024-07-19

**Authors:** Liying Zhai, Haihong Gong, Wencheng Yu

**Affiliations:** 1 Department of Pulmonary and Critical Care Medicine, The Affiliated Hospital of Qingdao University, Qingdao, China

**Keywords:** interstitial lung diseases, smoking, fibrosis, emphysema, prognosis

## Abstract

**INTRODUCTION:**

The presence of emphysema is common in patients with interstitial lung disease (ILD), which is designated as combined pulmonary fibrosis and emphysema (CPFE). This study aimed to examine the association between smoking, emphysema, and fibrosis in ILD patients.

**METHODS:**

A total of 800 patients hospitalized for ILD at the affiliated hospital of Qingdao University, Shandong, Qingdao, China, from December 2012 to December 2020 were included in our retrospective cohort study. Participants were divided into CPFE and non-CPFE groups. The patients’ clinical presentations and radiographic and laboratory findings were reviewed and compared. The two groups were then divided and compared based on smoking status. Kaplan-Meier survival analysis with log-rank testing and multivariable Cox proportional hazards regression analysis were used to compare all-cause mortality.

**RESULTS:**

Emphysema was present in 188 (23.5%) ILD patients. Smoking was associated with increased odds of CPFE (adjusted odds ratio, AOR=2.13; 95% CI: 1.33–3.41, p=0.002). The CPFE patients had a comparable risk of death to non-CPFE patients (adjusted hazard ratio, AHR=0.89; 95% CI: 0.64–1.24, p=0.493). Smoking was not a risk prognostic factor in the whole group (AHR=1.34; 95% CI: 0.90–1.99, p=0.152) or the CPFE group (AHR=0.90; 95% CI: 0.43–1.86, p=0.771). However, a significant prognostic difference between smokers and non-smokers was found in the non-CPFE group (AHR=1.62; 95% CI: 1.02–2.58, p=0.042). In ILD patients, smoking pack-years were weakly correlated with total centrilobular emphysema (CLE) scores and total fibrosis scores (TFS), but not with total emphysema scores (TES); TFS were weakly correlated with TES.

**CONCLUSIONS:**

CPFE did not affect the prognosis of ILD. Smoking was a risk but not a prognostic factor for CPFE. However, smoking was associated with worse survival in non-CPFE patients. There was an intricate association among smoking, emphysema, and fibrosis in ILD patients.

## INTRODUCTION

Interstitial lung diseases encompass a group of wide and heterogeneous pulmonary parenchymal disorders characterized by inflammatory-fibrotic infiltration^1,2^. In 1990, Wiggins et al.^3^ first described the coexistence of emphysema and pulmonary fibrosis on computed tomography (CT). In 2005, the name ‘combined pulmonary fibrosis and emphysema’ (CPFE) emerged^4^. The incidence of such CPFE is reported in 35% of idiopathic pulmonary fibrosis (IPF) patients and 26–54% of patients with idiopathic interstitial pneumonia^5,6^. However, a variable impact of emphysema on the survival of ILD patients has been reported^4,7^. Smoking is a definite risk factor for emphysema. There are, however, controversial reports about the role of smoking status in the prediction of mortality in ILD. Some studies reported that never-smoking IPF patients showed poor prognosis compared with smoking IPF patients^8-11^. The roles of emphysema and smoking in ILD have not been elucidated, and we wish to ascertain if there is an association between the two or the three. While investigating the association of fibrosis, emphysema, and smoking, we present a detailed analysis to compare the identified CPFE phenotype with an ILD group without emphysema. This study also subdivides them according to smoking status and evaluates the clinical and radiological characteristics of both CPFE and non-CPFE patients separately.

## METHODS

### Study design and patient selection

This study retrospectively reviewed 800 patients hospitalized for ILDs at the affiliated hospital of Qingdao University, Shandong, Qingdao, China, from December 2012 to December 2020. All subjects had a confirmed multidisciplinary diagnosis of ILD according to guidelines^12-15^. CPFE was defined as combined pulmonary fibrosis and emphysema^5^. Subjects with confirmed or suspected malignancy, concurrent pulmonary infection, uncontrolled heart disease, sarcoidosis, occupational lung disease, or radiation pneumonitis were excluded from the study. Patients without chest CT scans for review or lost to follow-up were excluded. Subjects were eligible for study inclusion if they had a multidisciplinary diagnosis of ILD and baseline chest CT scans obtained within one year of ILD diagnosis. This study was approved by the affiliated hospital of Qingdao University Institutional Review Board, Shandong, Qingdao, China, and conformed to the tenets of the Declaration of Helsinki. All patients signed informed consent.

### Data collection

The medical records of each patient’s initial clinic visit were reviewed, including demographic characteristics, clinical symptoms and signs, comorbid conditions, smoking history, laboratory and chest CT imaging findings, ultrasonic cardiogram, and pulmonary function test. Blood samples were collected on an empty stomach in the early morning after hospitalization. Biochemical parameters were measured using the Beckman AU5811 automatic biochemical analyzer (Beckman Coulter Inc., Krefeld, Germany) with commercially available kits (Leadman Biochemistry Co., Beijing, China). Spirometry was performed using a Jaeger Medical spirometer (Jaeger, Omaha, NB, USA) per standard guidelines^16,17^.

### Chest CT evaluation

Two radiologists blinded to clinical and outcome data independently measured all the chest CT scans. Discrepant readings were re-evaluated by a third radiologist with the greatest experience in pulmonary imaging. Fibrosis and emphysema were evaluated using semi-quantitative image analysis^18-21^. Briefly, CT images were scored at five levels (the origin of great vessels, the main carina, the pulmonary venous confluence, halfway between the third and fifth sections, and immediately above the right hemidiaphragm) and disease extent was visually scored in each of the five CT sections (0: absent; 1: 1–25%; 2: 26–50%; 3: 51–75%; and 4: 76–100%). The total extent of fibrosis was calculated as the mean extent score in the five scored CT sections. The modified coarseness of reticular disease (MCRD) was also calculated in each of the five sections as follows: 0=normal; 1=ground-glass opacity alone; 2=fine intralobular fibrosis; 3=microcystic honeycombing (≤4 mm); and 4=macrocystic honeycombing (>4 mm). MCRD was estimated based on the summed score for all five levels. The total fibrosis score was the product of the total extent of fibrosis and MCRD. The degree of three visually defined emphysematous destruction phenotypes was scored in each of the five sections as follows: a) CLE: 1, trace CLE; 2, mild CLE; 3, moderate CLE; 4, confluent CLE; 5, advanced destructive emphysema (ADE). b) panlobular emphysema. c) paraseptal emphysema (PSE): 1, mild PSE; 2, substantial PSE^20^. The scores for the three phenotypes were the sum of products of extent of emphysema and degree of emphysema in each of the five sections. The total emphysema score was the summed score for the three phenotypes (Table 1). All CT images were reviewed at lung window settings (with a window center of -500 to -600 HU and a window width of 1600 HU). Mediastinal lymph nodes (MLNs) were assessed using soft tissue windows only (level 35 Hounsfield unit [HU], width 450 HU) based on the International Association for the Study of Lung Cancer (IASLC; Denver, CO, USA) nomenclature^22^. MLN measurements were provided by radiologists from the reformatted imaging data using virtual calipers, and enlargement was defined as short-axis diameters ≥10 mm^23,24^. Intra- and inter-observer agreement was assessed using Kappa (κ) statistics and intra-class correlation coefficients (ICC). The ICC and κ values were considered poor agreement if they were <0.4; moderate agreement if 0.4–0.59; substantial agreement if 0.6–0.79; and almost-perfect agreement if they were 0.8–1. The intra- and inter-observer agreement for measurements of chest CT features was greater than 0.8 for all variables assessed.

**Table 1:** Summary of the computed tomography scoring system to examine the association between smoking, emphysema, and fibrosis in interstitial lung disease hospitalized patients at the affiliated hospital of Qingdao University, Shandong, Qingdao, China from December 2012 to December 2020 (N=800)

*Category*	*Scores*					
	*0*	*1*	*2*	*3*	*4*	*5*
**Disease extent**	Absent	1–25%	26–50%	51–75%	76–100%	
**MCRD**	Normal	Ground-glass opacity alone	Fine intralobular fibrosis	Microcystic honeycombing (≤4 mm)	Macrocystic honeycombing (>4 mm)	
**CLE**	Normal	Trace CLE	Mild CLE	Moderate CLE	Confluent CLE	Advanced destructive emphysema (ADE)
**PSE**	Normal	Mild PSE	Substantial PSE			

All CT images were scored at five levels (the origin of great vessels, the main carina, the pulmonary venous confluence, halfway between the third and fifth sections, and immediately above the right hemidiaphragm). The total extent of disease was calculated as the mean extent score in the five scored CT sections. MCRD was estimated based on the summed score for all five levels. The total fibrosis score was the product of the total extent of fibrosis and MCRD. The scores for the emphysema phenotypes were the sum of products of the extent of emphysema and degree of emphysema in each of the five sections. Total emphysema score was the summed score for all the emphysema phenotypes (panlobular emphysema was not present in our cohort). MCRD: the modified coarseness of reticular disease. CLE: centrilobular emphysema. PSE: paraseptal emphysema.

### Follow-up and endpoint of the study

The primary endpoint of our study was transplant-free survival, defined as the time from the diagnosis of ILD to death or lung transplantation. Each patient was followed up until the occurrence of death, lung transplantation, end of the study period, or loss of follow-up. Follow-up time was censored on 14 December 2023.

### Statistical analysis

The ILD patient group was divided and compared according to whether the patients had emphysema. The CPFE and non-CPFE groups were then divided based on smoking status. Categorical variables were processed using the χ^2^ test. The Shapiro-Wilk W-test was used to assess the normality of the data. Student’s t-test and the Mann-Whitney test were used for parametric and non-parametric distribution variables, respectively. After adjusting for study covariates, multivariable logistic regression analyses were conducted to obtain adjusted odds ratios (AORs) and 95% confidence intervals (CIs) for factors related to CPFE in ILD patients. The multivariable models were determined by stepwise selection of pertinent patient characteristics considered biologically relevant and variables with p<0.1 in univariable analyses. The correlations between smoking pack-years and TFS and TES were evaluated using Spearman’s ρ test. Survival curves were estimated by the Kaplan-Meier method, and differences in survival were compared using the log-rank test. Multivariable Cox proportional hazard regression analysis adjusted for covariates (including age, sex, etc.) was used to calculate adjusted hazard ratios (AHRs) and their 95% CIs. The proportional hazards assumption in the Cox model was tested based on the Schoenfeld residuals, and all models evaluated passed this test. Analysis was performed using SPSS v26.0 (IBM Corporation, Armonk, NY, USA). All tests were two-tailed, and the statistical significance of the difference was set at 0.05.

## RESULTS

### Characteristics of patients with ILD, according to the presence of emphysema

Baseline characteristics of 800 patients with ILD included in this study are shown in Table 2. Emphysema was present in 188 (23.5%) patients. The CPFE participants were older, more frequently smoked, complained of sputum production, and had significantly lower body mass index (BMI) and more IPF subtypes compared to non-CPFE patients. In addition, increased white blood cell (WBC) and monocyte count, higher N-terminal pro-B-type natriuretic peptide (NT-proBNP) levels, longer left ventricular end-diastolic dimension (LVDd), left ventricular end-systolic dimension (LVDs) and right atrial short-axis diameter (RASD), decreased prealbumin, triglycerides, total cholesterol (TC) and high-density lipoprotein cholesterol (HDL-cholesterol) levels, and lower arterial hemoglobin oxygen saturation (SaO2) were found in the CPFE group compared with the non-CPFE group. Furthermore, the CPFE patients had significantly lower lung diffusion capacity for carbon monoxide/alveolar ventilation (DLco/VA) and forced expiratory volume in 1 sec/forced vital capacity (FEV1/FVC) rates, higher residual volume (RV) and total lung capacity (TLC), higher total fibrosis score and prevalence of mediastinal lymph node enlargement (MLNE) and coronary artery (CA) calcification than non-CPFE patients. However, survival was not significantly different between the two groups (69.4% vs 72.9%, p=0.382).

**Table 2:** Characteristics of patients with interstitial lung disease, according to the presence of emphysema, China, from 2012 to 2020 (N=800)

*Characteristics*	*Overall* *(N=800)* *Median (IQR)*	*Non-CPFE* *(N=612)* *Median (IQR)*	*CPFE* *(N=188)* *Median (IQR)*	*p*
**Male,** n (%)	467 (58.4)	300 (49.0)	167 (88.8)	<0.001
**Age** (years)	65.00 (59.00–72.00)	65.00 (57.75–72.00)	67.00 (62.00–73.00)	0.001
**BMI** (kg/m^2^), mean ± SD	24.76 ± 3.37	24.98 ± 3.29	24.06 ± 3.58	<0.001
**Length of stay** (days)	10.50 (7.00–15.00)	11.00 (7.00–15.00)	9.00 (7.00–13.25)	0.026
**Time to diagnosis** (days)	180.0 (30.0–730.0)	135.0 (40.0–730.0)	240.0 (30.0–1095.0)	0.290
**Smoker,** n (%)	370 (46.2)	227 (37.1)	143 (76.1)	<0.001
**Sputum production,** n (%)	479 (59.9)	348 (56.9)	131 (69.7)	0.002
**Comorbidity,** n (%)				
Hypertension	206 (25.8)	162 (26.5)	44 (23.4)	0.446
Diabetes	120 (15.0)	99 (16.2)	21 (11.2)	0.102
CVD	117 (14.6)	91 (14.9)	26 (13.8)	0.814
**Subtypes of ILDs,** n (%)				0.018
IPF	308 (38.5)	219 (35.8)	89 (47.3)	
CTD-ILD	262 (32.8)	210 (34.3)	52 (27.7)	
NSIP	188 (23.5)	153 (25.0)	35 (18.6)	
Other	42 (5.2)	30 (4.9)	12 (6.4)	
**Laboratory findings**				
WBC count (×10^9^/L)	7.11 (5.66–8.93)	6.92 (5.53–8.90)	7.50 (6.07–8.94)	0.032
Neutrophil count (×10^9^/L)	4.20 (3.09–5.86)	4.14 (3.04–5.85)	4.31 (3.34–5.89)	0.258
Lymphocyte count (×10^9^/L)	1.95 (1.48–2.53)	1.94 (1.45–2.48)	2.00 (1.56–2.68)	0.058
Monocyte count (×10^9^/L)	0.54 (0.41–0.69)	0.53 (0.40–0.68)	0.57 (0.43–0.72)	0.019
Prealbumin (mg/L)	241.2 (189.0–300.6)	245.3 (193.7–301.8)	218.3 (179.0–289.8)	0.011
Triglyceride (mmol/L)	1.23 (0.90–1.83)	1.28 (0.93–1.90)	1.08 (0.82–1.58)	0.002
Total cholesterol (mmol/L)	4.80 (4.08–5.68)	4.86 (4.14–5.74)	4.66 (3.89–5.46)	0.027
HDL-cholesterol (mmol/L)	1.18 (1.00–1.44)	1.21 (1.02–1.46)	1.10 (0.98–1.34)	0.007
LDL-cholesterol (mmol/L)	2.92 (2.32–3.48)	2.94 (2.35–3.52)	2.82 (2.22–3.40)	0.141
SaO2 (%)	0.96 (0.94–0.97)	0.96 (0.94–0.97)	0.95 (0.93–0.97)	0.007
NT-proBNP (ng/L)	146.5 (52.0–301.0)	130.3 (49.1–215.1)	198.60 (104.5–490.8)	0.004
**Echocardiography**				
EF (%)	61.00 (60.00–63.00)	61.00 (60.00–63.00)	61.00 (60.00–63.00)	0.265
PASP (mmHg)	30.00 (25.25–38.00)	30.00 (26.00–37.00)	30.00 (25.00–39.00)	0.443
LVDd (cm)	4.50 (4.20–4.70)	4.40 (4.20–4.70)	4.50 (4.30–4.80)	0.005
LVDs (cm)	2.90 (2.70–3.10)	2.90 (2.70–3.00)	3.00 (2.80–3.10)	0.002
RASD (cm)	3.30 (3.00–3.50)	3.20 (3.00–3.50)	3.30 (3.10–3.60)	0.039
**Pulmonary function test**				
FEV1/FVC (%pred)	107.0 (101.0–113.0)	108.0 (102.0–114.0)	105.0 (97.0–110.0)	<0.001
TLC (%pred)	75.00 (61.00–87.00)	73.00 (59.50–84.00)	82.00 (69.90–91.65)	<0.001
RV (%pred)	74.00 (61.00–89.00)	72.00 (60.00–87.10)	81.00 (65.00–96.25)	0.002
RV/TLC (%pred)	98.00 (87.00–110.00)	97.00 (87.00–110.00)	98.00 (88.00–109.00)	0.961
DL_CO_/VA (%pred)	92.0 (76.6–107.0)	96.0 (81.8–111.0)	76.0 (63.5–92.5)	<0.001
**CT findings**				
MLNE, n (%)	313 (39.1)	220 (35.9)	93 (49.5)	0.001
Total fibrosis score	19.60 (11.00–33.00)	18.00 (10.80–30.80)	24.00 (14.00–40.00)	<0.001
Aortic calcification, n (%)	485 (60.6)	344 (56.2)	141 (75.0)	<0.001
CA calcification, n (%)	345 (43.1)	238 (38.9)	107 (56.9)	<0.001
Definite UIP, n (%)	300 (37.5)	202 (33.0)	98 (52.1)	<0.001
**Follow-up**				
PPF, n (%)	129 (16.1)	101 (16.5)	28 (14.9)	0.610
AE-ILD, n (%)	200 (30.8)	149 (30.0)	51 (33.3)	0.425
Survival time (months)	32.00 (17.00–56.00)	33.00 (16.25–57.00)	32.00 (18.00–52.00)	0.609
Overall survival, n (%)	519 (72.1)	399 (72.9)	120 (69.4)	0.382

Categorical variables were processed using the χ^2^ test. Student’s t-test and the Mann-Whitney test were used for parametric and non-parametric distribution continuous variables, respectively. ILD: interstitial lung disease. CPFE: combined pulmonary fibrosis and emphysema. IQR: interquartile range. BMI: body mass index. SD: standard deviation. CVD: cardiovascular disease. IPF: idiopathic pulmonary fibrosis. CTD-ILD: connective tissue disease-associated interstitial lung disease. NSIP: non-specific interstitial pneumonia. WBC: white blood cell. HDL-cholesterol: high density lipoprotein cholesterol. LDL-cholesterol: low density lipoprotein cholesterol. SaO2: arterial hemoglobin oxygen saturation. NT-proBNP: N-terminal pro B-type natriuretic peptide. EF: ejection fraction. PASP: pulmonary arterial systolic pressure. LVDd: left ventricular end-diastolic dimension. LVDs : left ventricular end-systolic dimension. RASD: right atrial short-axis diameter. FEV1: forced expiratory volume in 1 sec. FVC: forced vital capacity. RV: residual volume. TLC: total lung capacity. DLCO: lung diffusion capacity for carbon monoxide. VA: alveolar ventilation. CT: computed tomography. MLNE: mediastinal lymph node enlargement. CA: coronary artery. UIP: usual interstitial pneumonia. PPF: progressive pulmonary fibrosis. AE-ILD: acute exacerbation of interstitial lung disease.

Multivariable logistic regression analysis was performed to show that age (AOR=1.02; 95% CI: 1.01–1.04, p=0.011), male (AOR=4.63; 95% CI: 2.56–8.36, p<0.001), smoking (AOR=2.13; 95% CI: 1.33–3.41, p=0.002), and MLNE (AOR=1.51; 95% CI: 1.06–2.16, p=0.024) were associated with increased odds of CPFE in ILD patients. Spearman’s correlation analysis showed that smoking pack years were weakly correlated with total CLE scores (ρ=0.19, p=0.010) and TFS (ρ=0.21, p<0.001) but not with TES (ρ=0.14, p=0.051). TFS were weakly correlated with TES (ρ=0.38, p<0.001).

### Comparison of characteristics based on smoking status in CPFE and non-CPFE patients

In the CPFE group, we observed that smokers had higher CLE scores (5.00 vs 3.00, p=0.028) and more frequently experienced progression of emphysema on chest CT scans (34.5% vs 8.3%, p=0.002) compared to non-smokers. The other characteristics, including prognosis and demographic, clinical, laboratory, and chest CT imaging findings, exhibited no significant differences between smokers and non-smokers.

In the non-CPFE group, we discovered that smokers had increased WBC count, LVDd, RASD, and TFS, decreased DLco/VA, triglycerides, TC, and HDL-cholesterol levels, and higher prevalence of IPF, MLNE, and CA calcification than non-smokers. Intriguingly, smokers in non-CPFE patients exhibited a worse prognosis compared to non-smokers, including shorter survival time (29 vs 34 months, p=0.026) and lower survival rate (64% vs 78%, p=0.001). See Supplementary file Table S1 for details.

### Survival analysis of smoking behaviors in ILD patients stratified by the presence of emphysema

**Figure 1:**
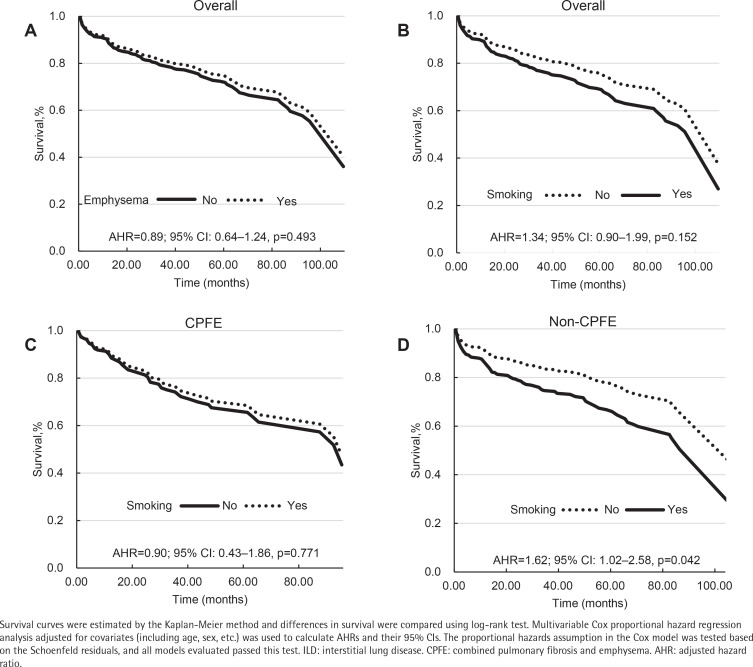
Survival analysis of smoking behaviors in ILD patients stratified by the presence of emphysema, China, 2012–2020 (N=800)

Our study utilized Kaplan-Meier survival analysis with log-rank testing and multivariable Cox proportional hazards regression analysis to determine that CPFE patients had a comparable risk of death to non-CPFE patients in the whole cohort (AHR=0.89; 95% CI: 0.64–1.24, p=0.493). Smoking was also not a risk prognostic factor in the whole (AHR=1.34; 95% CI: 0.90–1.99, p=0.152) or the CPFE group (AHR=0.90; 95% CI: 0.43–1.86, p=0.771). However, a significant prognostic difference between smokers and non-smokers was found in the non-CPFE group (AHR=1.62; 95% CI: 1.02–2.58, p=0.042) (Figure 1).

## DISCUSSION

The frequency of CPFE in ILD patients was reported to range from 8 to 67%^5^. In our cohort, emphysema was present in 23.5% of patients. Consistent with our knowledge that smoking was a key environmental risk factor and sputum production was the typical characteristic of chronic obstructive pulmonary disease (COPD)^25^, the CPFE patients had a higher prevalence of smoking and sputum production. The increased WBC and monocyte count in CPFE patients also revealed that COPD was a chronic inflammatory disease^26^. Moreover, the CPFE participants had significantly lower BMI and decreased prealbumin, triglyceride, TC, and HDL-cholesterol levels than non-CPFE patients. This suggested that the combination of emphysema may lead to greater physical exertion in ILD patients^27^. We also observed that CPFE patients had higher NT-proBNP level^28^, longer LVDd, LVDs, and RASD, and more CA calcification, which indicated that emphysema may exacerbate the heart burden of ILD patients. Echoing published literature^27,29^, the CPFE group had significantly higher RV and TLC and lower FEV1/FVC and DLCO/VA rates than the non-CPFE group.

Studies have reported that males, smoking, FEV1/FVC, and DLCO/VA were associated with CPFE^29,30^. Our analysis revealed that age, male, smoking, and MLNE were independent predictive factors for CPFE in ILD patients.

This study also divided the CPFE and non-CPFE groups according to smoking status. In the CPFE group, results showed that smoking was associated with higher CLE scores and emphysema progression on CT scans. On the other hand, we observed numerous resemblances between smokers and non-smokers in the non-CPFE group, as well as between CPFE and non-CPFE in the whole cohort (including increased WBC count, LVDd, RASD and TFS, decreased DLco/VA, triglycerides, TC and HDL-cholesterol levels, and higher prevalence of IPF, MLNE, and CA calcification). These further demonstrated the key role of smoking in the development of emphysema based on fibrosis.

There have been controversial reports on how smoking and emphysema affected survival in ILDs^4,7-11,30-32^. Our survival analysis indicated that CPFE did not affect the prognosis of ILD. Smoking was also not a prognostic factor in the whole or the CPFE groups. However, smoking was a risk prognostic factor in the non-CPFE group. These findings revealed that smoking had adverse effects on ILD prognosis, which may be confused by the combination of emphysema.

Correlation analysis showed that smoking pack-years were weakly correlated with total CLE scores and TFS but not with TES. TFS were weakly correlated with TES. Smoking, emphysema, and fibrosis are associated with each other in ILD patients.

### Limitations

Our study’s main limitation is its retrospective and non-causal single-center design. Second, while most CT scans were high-resolution ILD protocol images, a few patients only had standard chest CT scans. Third, we did not consider other possible confounding factors, such as secondhand smoke exposure. Finally, our analysis results have limited generalizability to other countries. Therefore, further multi-center prospective randomized controlled clinical studies are needed.

## CONCLUSIONS

Smoking is a risk factor for CPFE. CPFE did not affect the prognosis of ILD. Smoking was not a prognostic factor in the whole ILD or the CPFE groups but in the non-CPFE group. In ILD patients, total fibrosis scores were weakly correlated with total emphysema scores; smoking pack-years were weakly correlated with total CLE scores and total fibrosis scores but not with total emphysema scores. There was an intricate association between smoking, emphysema, and fibrosis in ILD patients.

## Supplementary Material



## Data Availability

The data supporting this research are available from the authors on reasonable request.
