# Supplementary material for: The link between smoking, emphysema, and fibrosis: A retrospective cohort study
Source: Tob Induc Dis. 2024 Jul 19;22:10.18332/tid/190689. doi: 10.18332/tid/190689 (PMC11258697; doi:10.18332/tid/190689)
Supplement: Supplementary file 1 [file TID-22-132-s1.pdf]

Table S1 Characteristics of ILD patients without emphysema, according to the status of smoking, China, 2012–2020 (N=800)

	Overall (n=612) median [IQR]	Non-smokers (n=385) median [IQR]	Smokers (n=227) median [IQR]	P value
Age, years	65.00 [57.75, 72.00]	65.00 [56.00, 71.00]	65.00 [59.50, 72.00]	0.207
Male, n (%)	300 (49.0)	87 (22.6)	213 (93.8)	<0.001
Length of stay, days	11.00 [7.00, 15.00]	12.00 [7.00, 16.00]	10.00 [7.00, 14.00]	0.010
Time to diagnosis, days	135.00 [40.00, 730.00]	90.00 [30.00, 730.00]	182.50 [60.00, 730.00]	0.006
Subtypes of ILDs, n (%)				
CTD-ILD	210 (34.3)	164 (42.6)	46 (20.3)	<0.001
IPF	219 (35.8)	93 (24.2)	126 (55.5)	
NSIP	153 (25.0)	106 (27.5)	47 (20.7)	
Others	30 (4.9)	22 (5.7)	8 (3.5)	
Laboratory findings				
WBC count, $\times 10^9$/L	6.92 [5.53, 8.90]	6.80 [5.43, 8.78]	7.21 [5.84, 9.25]	0.037
Lymphocyte count, $\times 10^9$/L	1.94 [1.45, 2.48]	1.79 [1.35, 2.31]	2.07 [1.61, 2.62]	<0.001
Triglyceride, mmol/L	1.28 [0.93, 1.90]	1.38 [0.99, 2.05]	1.17 [0.85, 1.73]	<0.001
Total cholesterol, mmol/L	4.86 [4.14, 5.74]	4.97 [4.19, 5.95]	4.71 [3.97, 5.58]	0.014
HDL-cholesterol, mmol/L	1.21 [1.02, 1.46]	1.23 [1.04, 1.49]	1.15 [0.98, 1.44]	0.040
LDL-cholesterol, mmol/L	2.94 [2.35, 3.52]	2.98 [2.40, 3.56]	2.91 [2.25, 3.47]	0.145
Echocardiography				
EF, %	61.00 [60.00, 63.00]	61.00 [60.00, 64.00]	61.00 [60.00, 63.00]	0.062
PASP, mmHg	30.00 [26.00, 37.00]	30.00 [26.00, 37.00]	30.00 [25.00, 38.00]	0.873
LVDd, cm	4.40 [4.20, 4.70]	4.40 [4.20, 4.60]	4.50 [4.30, 4.73]	<0.001
LVDs, cm	2.90 [2.70, 3.00]	2.90 [2.60, 3.00]	2.90 [2.70, 3.10]	0.177
RASD, cm	3.20 [3.00, 3.50]	3.20 [3.00, 3.40]	3.30 [3.10, 3.60]	<0.001
Pulmonary function test				
FEV1/FVC (%predicted)	108.0 [102.0, 114.0]	108.0 [102.4, 113.4]	109.0 [102.0, 114.7]	0.518
RV/TLC (%predicted)	97.00 [87.00, 110.00]	99.75 [89.30, 112.00]	95.00 [86.00, 106.00]	0.007
DL_{CO}/VA (%predicted)	96.00 [81.75, 111.00]	98.85 [84.00, 112.25]	92.50 [78.75, 106.00]	0.046
CT findings				
MLNE, n (%)	220 (35.9)	125 (32.5)	95 (41.9)	0.023
Total fibrosis score	18.00 [10.80, 30.80]	16.00 [10.00, 25.20]	22.40 [14.00, 40.00]	<0.001
Aortic calcification, n(%)	344 (56.2)	194 (50.4)	150 (66.1)	<0.001
CA calcification, n(%)	238 (38.9)	123 (31.9)	115 (50.7)	<0.001
Definite UIP, n(%)	202 (33.0)	78 (20.3)	124 (54.6)	<0.001
Follow-up				
PPF	101 (16.5)	73 (19.0)	28 (12.3)	0.599
AE-ILD, n(%)	149 (30.0)	93 (29.2)	56 (31.3)	0.684
Survival time, months	33.00 [16.25, 57.00]	34.00 [19.00, 58.00]	29.00 [14.00, 54.25]	0.026
Overall survival, n(%)	399 (72.9)	273 (78.0)	126 (64.0)	0.001

Median (IQR), or n (%) are presented for parameters. Categorical variables were processed using the χ^2 test. Mann-Whitney test were used for continuous variables.

ILD = interstitial lung disease; IQR = interquartile range; IPF = idiopathic pulmonary fibrosis; CTD-ILD = connective tissue disease-associated interstitial lung disease; NSIP = non-specific interstitial pneumonia; WBC = white blood cell; HDL-cholesterol = high density lipoprotein cholesterol; LDL-cholesterol = low density lipoprotein cholesterol; EF = ejection fraction; PASP = pulmonary arterial systolic pressure; LVDd = left ventricular end-diastolic dimension; LVDs = left ventricular end-systolic dimension; RASD = right atrial short-axis diameter; FEV1 = forced expiratory volume in 1 sec; FVC = forced vital capacity; RV = residual volume; TLC = total lung capacity; DL_{CO} = lung diffusion capacity for carbon monoxide; VA = alveolar ventilation; CT = computed tomography; MLNE = mediastinal lymph node enlargement; CA = Coronary artery; UIP = usual interstitial pneumonia; PPF = progressive pulmonary fibrosis; AE-ILD = acute

exacerbation of interstitial lung disease.

©2024 Zhai L. et al.
